# Haploid and Sexual Selection Shape the Rate of Evolution of Genes across the Honey Bee (*Apis mellifera* L.) Genome

**DOI:** 10.1093/gbe/evac063

**Published:** 2022-05-12

**Authors:** Garett P. Slater, Amy L. Dapper, Brock A. Harpur

**Affiliations:** Department of Entomology, Purdue University, 901 W State St., Lafayette, Indiana 47905, USA; Department of Biological Sciences, Mississippi State University, 219 Harned Hall, 295 Lee Blvd, Mississippi State, Mississippi 39762, USA; Department of Entomology, Purdue University, 901 W State St., Lafayette, Indiana 47905, USA

**Keywords:** haploid selection, sexual selection, honey bees

## Abstract

Many species have separate haploid and diploid phases. Theory predicts that each phase should experience the effects of evolutionary forces (like selection) differently. In the haploid phase, all fitness-affecting alleles are exposed to selection, whereas in the diploid phase, those same alleles can be masked by homologous alleles. This predicts that selection acting on genes expressed in haploids should be more effective than diploid-biased genes. Unfortunately, in arrhenotokous species, this prediction can be confounded with the effects of sex-specific expression, as haploids are usually reproductive males. Theory posits that, when accounting for ploidal- and sex-specific expression, selection should be equally efficient on haploid- and diploid-biased genes relative to constitutive genes. Here, we used a multiomic approach in honey bees to quantify the evolutionary rates of haploid-biased genes and test the relative effects of sexual- and haploid-expression on molecular evolution. We found that 16% of the honey bee’s protein-coding genome is highly expressed in haploid tissue. When accounting for ploidy and sex, haploid- and diploid-biased genes evolve at a lower rate than expected, indicating that they experience strong negative selection. However, the rate of molecular evolution of haploid-biased genes was higher than diploid-based genes. Genes associated with sperm storage are a clear exception to this trend with evidence of strong positive selection. Our results provide an important empirical test of theory outlining how selection acts on genes expressed in arrhenotokous species. We propose the haploid life history stage affects genome-wide patterns of diversity and divergence because of both sexual and haploid selection.

**Significance**

A common feature of most eukaryotic species is the presence of a separate haploid and diploid phase. Theory predicts that each phase should experience the effects of natural selection and drift differently. In a haploid phase, for example, all deleterious alleles are exposed to selection whereas in diploid phases those same alleles can be masked by homologous alleles. Unfortunately, for haplodiploid animal species, this prediction can be confounded with sexual selection as haploids are usually reproductive males. Here, we develop theory to predict how haploid- and sex-specific genes should evolve. We then use honey bees as a model to empirically test our predictions. We found that at least 16% of the honey bee’s protein-coding genome is highly expressed in haploid tissue. When accounting for ploidy and sex, there are significant differences in the molecular rates of evolution of haploid-biased genes relative to other diploid-biased and constitutively expressed genes sets. Despite this, haploid-biased genes tend to have much lower evolutionary rates than predicted. However, haploid-biased sperm storage genes are an exception. Our results provide an important empirical test of theory outlining how selection acts on genes expressed in arrhenotokous species. We propose the haploid life-history stage affects genome-wide patterns of diversity and divergence because of both sexual and haploid selection.

## Introduction

Population genetics is driven by the need to understand how and why allelic variation is distributed within a population. Many of the foundational genetic models assume populations consist of either diploids or haploids (e.g., Crow and Kimura 1970; Gillespie 1973; Kirzhner and Lyubich 1997). These models have been invaluable in helping us understand how allele frequencies change through time and how ploidy can influence selection and drift. For example, comparisons of models between haploid and diploid systems have provided evidence of a “masking effect”: all alleles with fitness effects in haploids experience selection directly, whereas those alleles in diploids may be “masked” from selection if acting recessively (Crow and Kimura 1965). While classic haploid- and diploid-specific models have been useful to understand evolutionary processes, they fail to consider populations with co-existing or alternating diploid and haploid life history stages with selection acting on each stage.

There is, of course, some historic precedence for modeling allele frequency changes in mixed-ploidy species (Hartl 1972; Crozier 1977; Otto and Goldstein 1992). Hartl (1972) demonstrated that, with all else equal, alleles in haploid-expressed genes in an arrhenotokous population can fix at a rate one-third faster than those same alleles in diploid-expressed genes. Historic models have provided a fruitful framework for more recent modeling attempts to explore the genetic evolution of arrhenotokous populations and how the haploid phase impacts evolution (Kondrashov and Crow 1991; Gerstein et al. 2011; Otto et al. 2015; Scott et al. 2018; Raices et al. 2019). Models have found that having a free-living haploid stage can lead to dramatically different evolutionary dynamics compared with strictly diploid or haploid populations (Hedrick and Parker 1997; Dapper and Wade 2016; Bessho and Otto 2017). One notable feature is the effect of selection. Selection acting on haploid-biased or haploid-limited genes (haploid selection) is predicted to greatly influence their levels of standing variation and potentially loci linked to them (Immler et al. 2012; Dapper and Wade 2016, 2020). Selection on haploid loci may even lead to recombination rate variation between haploid- and diploid-biased genes (Lenormand and Dutheil 2005).

Population genetic models and empirical studies for arrhenotokous systems have been informative to our understanding of how haploid-expressed genes evolve (e.g., Clarke et al. 2004; Arunkumar et al. 2013; Szövényi et al. 2013). Unfortunately, these models may not be entirely appropriate null predictions for most arrhenotokous animals because of confounding sex-specific expression. All Hymenoptera—potentially the most speciose order of insects (Forbes et al. 2018)—are arrhenotokous. Unfertilized hymenopteran eggs develop into males, and fertilized eggs usually develop into females (Harpur et al. 2013; Slater et al. 2020). Males are almost exclusively reproductive (Michener 2000). Most male-expressed genes will therefore experience haploid selection but will also experience varying degrees of sexual selection. Across most species, reproductive genes have elevated levels of sequence divergence when compared with genes not involved in reproduction (Swanson and Vacquier 2002; Panhuis et al. 2006; Vicens et al. 2014). For example, in *Capsella grandiflora*, pollen-specific genes (375 total) have a higher rate of adaptation than sporophyte-specific genes (Arunkumar et al. 2013). Elevated sequence divergence in reproductive genes is frequently attributed to strong sexual selection but may also be explained by relaxed selection (Meisel 2011; Dapper and Wade 2016, 2020; Mank 2017). In the case of arrhenotokous species, sex-biased expression could be conflated with haploid expression and, as we demonstrate in our companion paper, these two processes can have opposing effects on genomic evolution (Dapper et al. 2022). This is because a male-specific autosomal allele will always be maternally inherited and will not have experienced selection in the previous generation, while a female-specific allele will have a 50% chance of being paternally inherited and thus a comparatively higher chance of being exposed to selection in the previous generation. Our model (Dapper et al. 2022) proposes that, when accounting for ploidal environment and sex-specific expression, selection is less efficient on haploid- and diploid-biased genes relative to constitutively expressed genes. Therefore, our null expectation is that both haploid- and diploid-biased genes will have elevated levels of polymorphism and divergence when compared with constitutively expressed genes and that they will not differ significantly from each other. Furthermore, genes involved in postcopulatory sexual selection (PCSS) (e.g., some genes expressed in male gonad) are predicted evolve at a rate scaled by the number of mates a female has and, in honey bees, should evolve at the same rate as haploid- and diploid-biased genes (Dapper et al. 2022).

Testing the influences of sexual and haploid selection and their interactions is possible within the hymenoptera where male genes are expressed throughout the animal’s life span and likely account for a large fraction of the protein-coding genome. In the Western honey bee (*Apis mellifera* L.; henceforth honey bee), haploid males take 24 days to develop from eggs, have an adult lifespan between of up to approximately 40 days, and their sperm survives inside a queen’s spermatheca for her entire lifespan of approximately three years (Page and Peng 2011; Slater et al. 2021). This likely means that many genes in hymenopteran species experience some degree of haploid- and sexual selection, perhaps many more than pollen-specific genes in plants and sperm-specific genes in diploid animal models (Joseph and Kirkpatrick 2004). Honey bees are especially useful models. Honey bees have a high-quality chromosome-level reference genome (Wallberg et al. 2019), population genomic (Harpur, Kent, et al. 2014), and transcriptomic data sets (Chen et al. 2012) that make testing specific population genomic questions tractable. These data sets have been developed and used for studies on the evolution and diversification of genes expressed in female castes (reproductive queens and nonreproductive workers) (Evans and Wheeler 1999; Harpur, Kent, et al. 2014; Wallberg et al. 2019) and in genes associated with female-expressed behaviors (Lattorff and Moritz 2013; Harpur, Chernyshova, et al. 2014). There has been considerably less attention on how genes expressed in males evolve. Here, we use whole-genome resequencing data of honey bees (Harpur, Kent, et al. 2014) paired with existing (Chen et al. 2012) and new RNA-sequencing data sets of haploid and diploid larval, gonadal, and somatic tissues to estimate rates of adaptation, genetic diversity, and recombination to study the long-term impact of haploid and sexual selection on genome evolution. Specifically, we ask three major questions: what percentage of the honey bee genome experiences haploid selection, how is selection acting on male-biased genes and does this align with our theoretical predictions, and how has selection on male-biased genes impacted genetic diversity and recombination rates?

## Results

### Many Protein-Coding Genes in the Honey Bee Genome Experience Haploid Selection

**Fig. 1.**
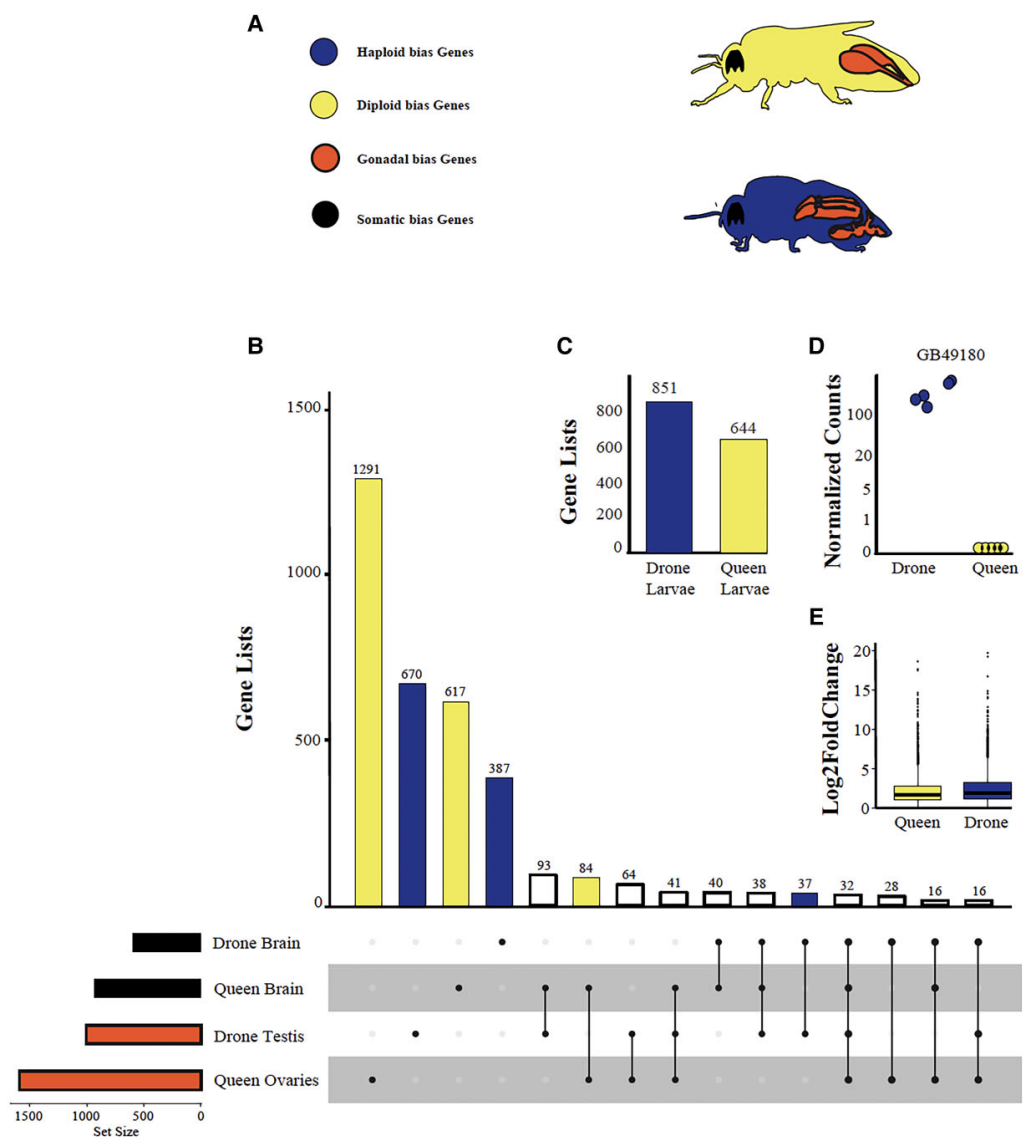
Differential expression of haploid- and diploid-expressed genes. (*A*) We dissected gonadal and somatic tissues from haploids (males) and diploids (female) and categorized them based on ploidy and tissue type. (*B*) UpSet Plot of up-regulated genes (where n = number of genes in a comparison and dots represent sample origin) among tissues in haploids and diploid adults and (*C*) for fifth instar larva. (*D*) An example of a haploid-biased gene identified by our analysis. GB49180, a cysteine-rich secretory protein, is highly expressed in male larval tissue but has limited to no expression in females. We predict that genes such as this are the most likely to experience selection in the haploid state.

We compared gene expression among outbred drone (haploid) and queen (diploid) brains and gonads (See Methods) as well as outbred haploid and diploid larvae obtained from a separate study (He et al. 2019). We identified genes with up-regulated expression in male or female tissues using pairwise comparisons among tissues within adults and larvae, separately (see Methods). In total, we identified 4,913 genes up-regulated in a single tissue or sex. The majority of these (1,291) were found within the queen’s gonads (fig. 1). In total, we discovered that at least 15.7% of the honey bee’s annotated, protein-coding genes (1,945/12,374) are up-regulated in at least one male tissue and 5.41% only in male testes (670/12,374) (fig. 1). We propose that any gene that is significantly up-regulated within males is likely to experience selection only or most strongly in the haploid state (i.e., experience haploid-selection and be haploid-biased) and they can be compared to genes that are significantly up-regulated within females (i.e., experience selection in the diploid state and are diploid-biased). Similarly, we propose that genes expressed in gonadal tissue are more likely to experience sexual selection than genes expressed in somatic tissues and larvae. Using these gene sets, we can test predictions outlined above and in Dapper et al. (2022).

### Haploid-Biased Genes Have Higher GC Content than Diploid-Biased Genes but Have Similar Recombination Rates, on Average

**Fig. 2.**
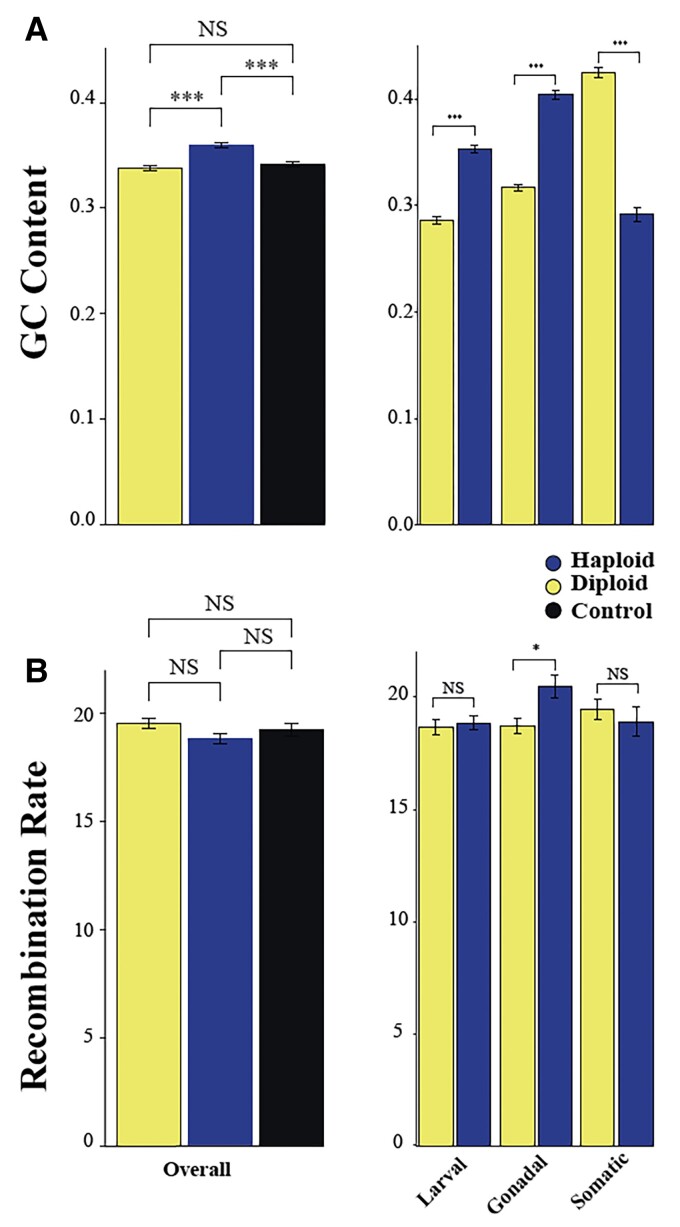
GC content and recombination rate in haploid-biased genes. (*A*) GC content was highest in haploid expressed genes. GC content was also higher in haploids in each tissue comparison, except for somatic tissue. (*B*) Recombination rate was higher in haploid expressed genes, but it was insignificant. Recombination rate did not differ in tissue comparisons, except gonadal tissue did have a higher recombination rate in haploid-biased genes. ***P < 0.0001, **P < 0.001, *P < 0.05. NS, nonsignificance.

Prior to testing the above hypotheses, we controlled for potential confounds. GC content (Guanine-Cytosine content; the proportion of GC for a given locus) in the honey bee genome correlates with several population genomic statistics, most notably with levels of synonymous diversity and recombination rates (Kent et al. 2012; Liu et al. 2015). We found significant differences in GC content between male-biased versus constitutive genes (fig. 2*A*; analysis of variance [ANOVA] *F*_2,6911_ = 31.84, *P* < 0.0001) but not between female-biased and constitutive genes (fig. 2*A*; ANOVA *F*_2,6911_ = 31.84, *P* = 0.31). We also found significant differences in the GC content of genes expressed in males versus females (fig. 2*A*; ANOVA *F*_1,6911_ = 31.84, *P* < 0.0001). This pattern was consistent across pairwise comparisons of each tissue (fig. 2*A*; ANOVA *F*_5,4078_ = 194.8, *P* < 0.0001) with genes overexpressed in male gonadal tissue and haploid larvae having higher overall GC content than corresponding female, diploid, tissue. Because of this, we included GC content of each gene as a covariate in all subsequent analyses and reported both results (with and without the covariate) where necessary (see Methods). While we observed differences in GC content between sexes and among tissues, we did not observe any statistically significant differences in recombination rates overall (fig. 2*B*; ANOVA *F*_2,6911_ = 1.953, *P* = 0.142) nor when controlling for GC content. This pattern was also true of all tissue comparisons, except for gonadal tissue (fig. 2*B*; ANOVA *F*_5,4078_ = 2.427, *P* = 0.0184). When controlling GC-content, none of the tissue comparisons were significant. These same patterns hold for CpGO/E ratios (indicative of methylation) among tissues (ANOVA *F*_5,4078_ = 87.38, *P* < 0.0001). This is unsurprising given the high correlation between a gene’s GC content and CpGo/e (ratio of observed to expected CpG dinucleotides) (Pearsons *R* = 0.375, *t*-value = 26.177, degrees of freedom [df] = 4175, *P* < 0.0001).

### Haploid-Biased Genes have Higher Levels of Genetic Diversity and Experience Positive Selection More Frequently than Diploid-Biased Genes

**Fig. 3.**
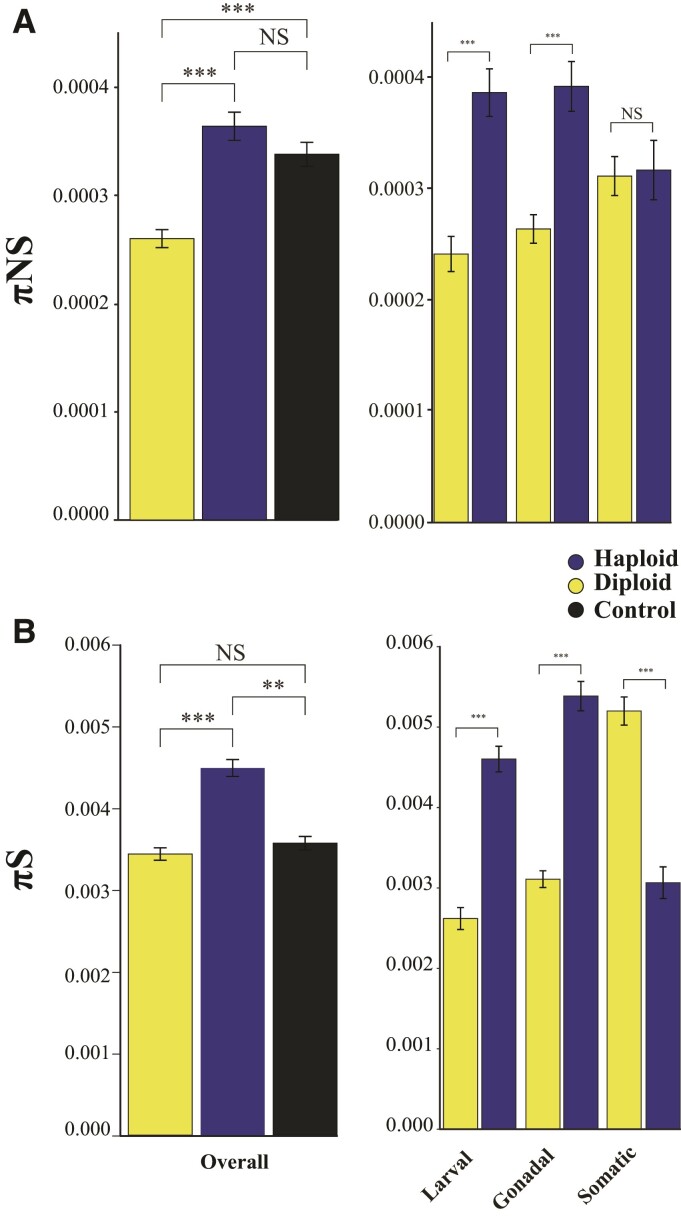
Genetic diversity. (*A*) We found haploid-biased genes had higher πNS compared with diploid-biased genes. πNS diversity was significantly higher for each tissue comparison in haploids except somatic tissue was insignificant. (*B*) Haploid-biased genes had higher πS. This remained true for tissue specific comparisons as haploid-biased genes had higher πS than their diploid counterparts, except for somatic tissue. ***P < 0.0001, **P < 0.001, *P < 0.05. NS, nonsignificance.

We predict that haploid- and diploid-biased genes will have equally elevated levels of standing variation in comparison with control genes (genes not differentially expressed among conditions; see Methods) overall and among homologous tissues (Dapper et al. 2022). We tested this hypothesis using both synonymous and nonsynonymous sites by estimating synonymous nucleotide diversity (πS) and nonsynonymous nucleotide diversity (πNS) for all genes (fig. 3). Overall, haploid-biased genes had higher πNS (fig. 3*A*; ANOVA *F*_2,6911_ = 41.83, *P* < 0.0001) when compared to diploid-biased genes and when controlling for GC content, but the haploid-biased genes were not significantly different from control genes. The haploid-biased genes also had higher πS (fig. 3*B*; ANOVA *F*_2,6911_ = 50.24, *P* < 0.0001) when compared with diploid- restricted and control genes and when controlling for GC content. Overall, both πNS (Log2 Fold β = 0.00003781, *P* < 0.0001) and πS (Log2 Fold β = 0.0006119, *P* < 0.0001) increased with greater haploid-biased expression. This pattern was largely consistent across pairwise comparisons of each tissue. Haploid-biased genes had higher πS and πNS relative to diploid-biased genes for every comparison except for πNS somatic tissue (πNS: ANOVA *F*_5,4078_ = 11.79, Tukey-HSD *P* = 0.999) (fig. 3). We also found constitutively expressed genes had a lower πS (fig. 3*B*; ANOVA *F*_2,6911_ = 50.24, *P* < 0.0001, Tukey-HSD *P* < 0.0001) and πNS (fig. 3*A*; ANOVA *F*_2,6911_ = 41.83, *P* < 0.0001, Tukey-HSD *P* < 0.0001) than haploid-biased genes, but constitutively expressed control genes did not differ from diploid-biased genes in both πS (Tukey-HSD *P* = 0.922) and πNS (Tukey-HSD *P* = 0.542). When controlling for GC content, we found somatic tissue was not significant for πS (analysis of covariance [ANCOVA] *F*_5,5283_ = 7.185, Tukey-HSD *P* = 0.999).

**Fig. 4.**
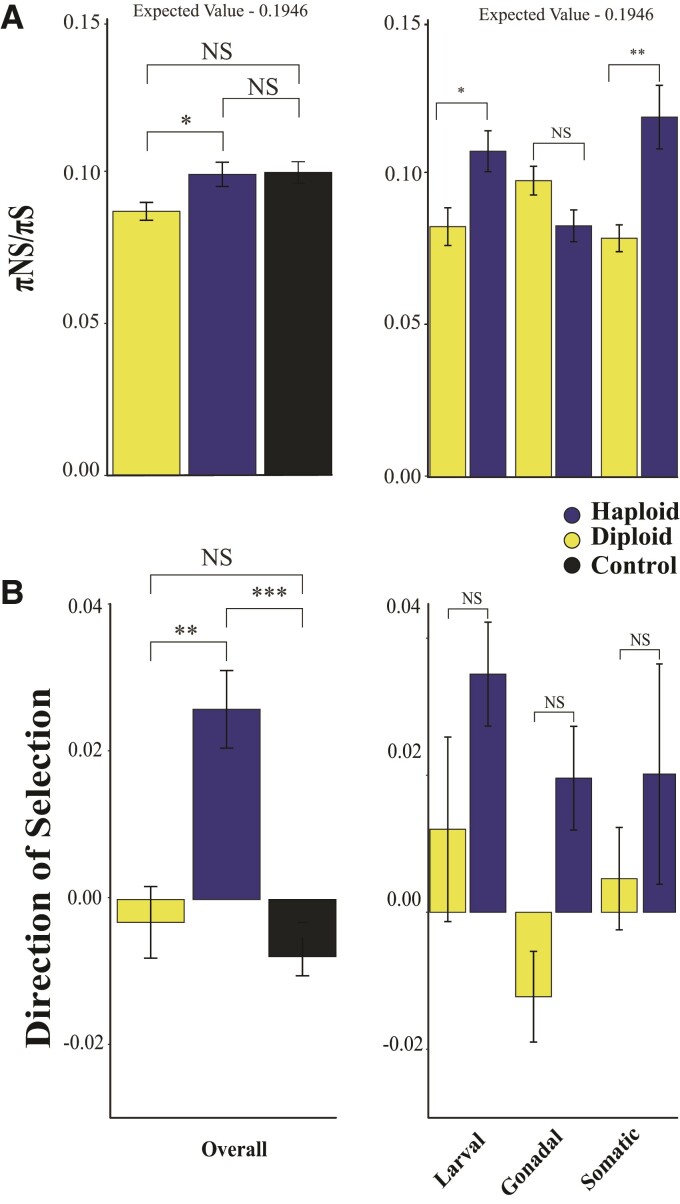
Selection. (*A*) We found that the πNS/πS ratio of haploid-biased genes was higher than diploid-biased genes. In the tissue comparisons, haploids had higher πNS/πS, except for gonadal tissue. (*B*) We found McDonald Kreitman DoS was higher for haploid-biased genes. DoS was higher for haploid-biased genes for all tissue comparisons, albeit they were all insignificant. Expected values predicted for sex-specific (haploid and diploid) genes as per Methods. ***P < 0.0001, **P < 0.001, *P < 0.05. NS, nonsignificance.

All else being equal, we expect that haploid expression will lead to an increase in the efficacy of selection (Gerstein et al. 2011; Dapper and Wade 2016; Immler and Otto 2018). However, the increase in the efficacy of selection due to haploidy will be offset by a similar reduction in the efficacy of selection because two-thirds of the copies of the genes are hidden from selection in diploid females (Dapper et al. 2022 and Supplemental). We therefore predict no differences in selection metrics between diploid- and haploid-biased genes and both sets should have evidence of relaxed selection relative to control genes. We tested this prediction using three such selection metrics. Firstly, we estimated πNS/πS as a proxy for selection within populations (Nelson et al. 2015). Considering sex-specific expression and ploidal environment, the null expectation is that πNS/πS for sex-biased (haploid and diploid) genes will be twice that observed among constitutive genes (0.1946 given an observed πNS/πS of 0.097 for constitutive genes) (See: Dapper and Wade 2016; Dapper et al. 2022). Both haploid- (average = 0.102) and diploid-biased (average = 0.0899) genes had πNS/πS lower than expected and there was a small but significant difference between πNS/πS of haploid-biased genes and diploid-biased genes overall (fig. 4*A*; ANOVA *F*_2,6911_ = 3.14, *P* = 0.0434) and across tissues, except for gonadal tissue (fig. 4*A*; ANOVA *F*_5,4078_ = 5.191, Tukey-HSD *P* = 0.415). Results were consistent across tissues when controlling for GC content (ANCOVA *F*_5,5283_ = 1.701 *P* = 0.131). Additionally, we found no significant relationship between πNS/πS and greater expression in haploid relative to diploid tissue overall (Log2 Fold β = −0.00196, *P* = 0.38).

Secondly, we estimated the Direction of Selection (DoS), a derivative of the McDonald–Kreitman test (McDonald and Kreitman 1991). DoS measures the direction and extent of selection on a given gene and is positive when there is evidence of adaptive evolution, zero when neutral, and negative when slightly deleterious mutations are segregating within a population (Stoletzki and Eyre-Walker 2011). We found that DoS was significantly higher for haploid-biased genes than diploid-biased genes, overall (fig. 4*B*; ANOVA *F*_2,6494_ = 12.3, *P* < 0.0001), and only diploid-biased genes were not significantly different from control genes. We found no significant differences in DoS among tissues. As the level of haploid expression increased relative to diploid expression, DoS increased significantly (Log2 Fold β = 0.00752, *P* = 0.008). There was also a higher proportion of haploid-biased genes with evidence of positive selection (genes with a significant McDonald–Kreitman test *P* < 0.05 and DoS > 1) relative to diploid-biased genes (haploid 6.59% diploid 3.76%; χ^2^ = 15.01, df = 1, *P* = 0.0001). Constitutively expressed control genes also had a lower proportion of genes with evidence of positive selection than both haploid-biased genes (haploid 6.59% control 0%; χ^2^ = 171.09, df = 1, *P* < 0.0001) and diploid-biased genes (diploid 3.76% control 0%; χ^2^ = 15.01, df = 1, *P* = 0.0001).

**Fig. 5.**
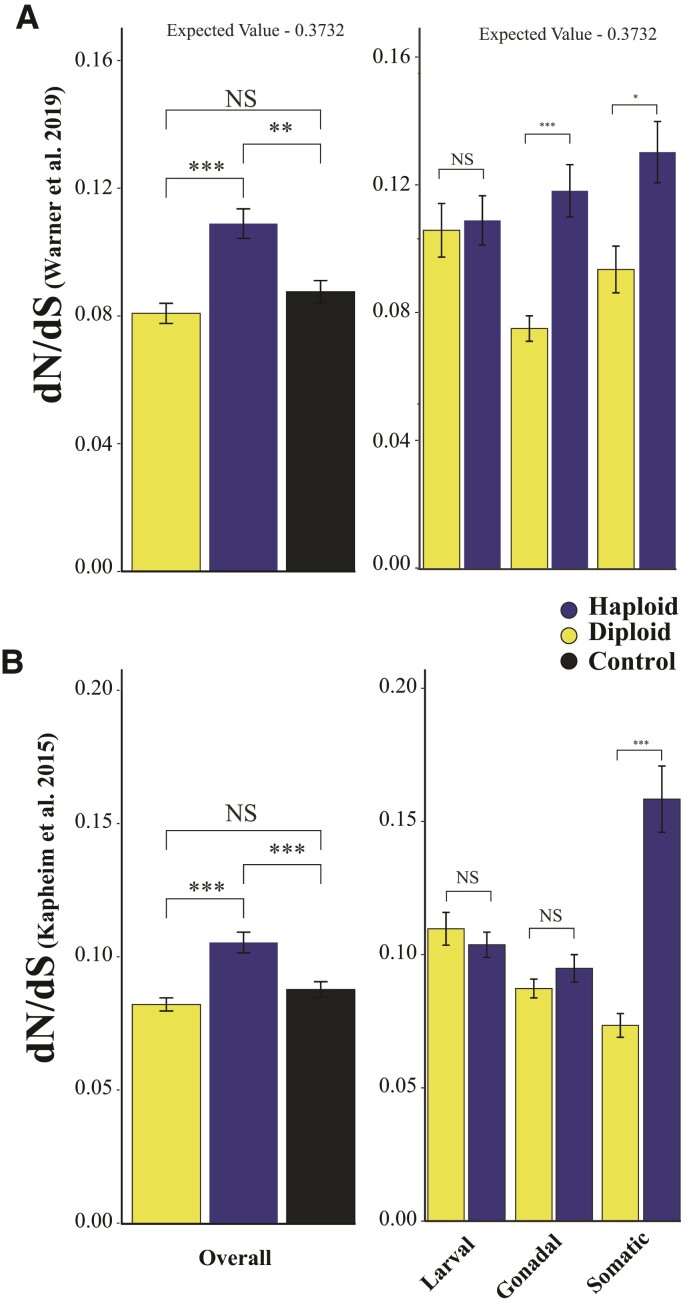
dN/dS. (*A*) As estimated by Warner et al. (2019), we found dN/dS in haploid-biased genes to be higher than diploid-biased genes. Haploid expressed genes also had a higher dN/dS in each tissue comparison. (*B*) In Kapheim et al. (2015), haploid-biased genes also had higher dN/dS overall. In each tissue comparison, haploid-biased genes had a higher dN/dS, except for larval tissue. Expected values predicted for sex-specific (haploid and diploid) genes as per Methods. ***P < 0.0001, **P < 0.001, *P < 0.05. NS, nonsignificance.

Thirdly, we compared dN/dS (the ratio of nonsynonymous codon-substitution rate to synonymous codons) as calculated previously (Kapheim et al. 2015; Warner et al. 2019). We used these two data sets independently to ensure our results were not biased by differences in the underlying phylogenies, ortholog identification, nor any other methodological variation. We predicted the average dN/dS for sex-biased (haploid and diploid) genes to be 0.3732 given an observed dN/dS of 0.092 for constitutive genes (see model in Methods and Dapper et al. 2022) and assuming the harmonic mean number of mates per female queen is 13.984 (*A. mellifera*) (Slater et al. 2021). We found both haploid- and diploid-biased genes had lower dN/dS than predicted across both datasets. Further, haploid-biased genes had higher dN/dS than diploid-biased genes in both datasets and higher than constitutively expressed genes (fig. 5; Warner: ANOVA *F*_2,2503_ = 12.53, *P* < 0.0001; Kapheim: ANOVA *F*_2,2503_ = 12.06, *P* < 0.0001). Among tissues, we found haploid-restricted genes had higher dN/dS than diploid-restricted genes in all comparisons except larval tissue for the Warner et al. (2019) dataset (fig. 5*A*; ANOVA *F*_5,1596_ = 9.376, Tukey-HSD *P* = 0.999) and both larval and gonadal tissue for the Kapheim et al. (2015) dataset (fig. 5*B*; ANOVA *F*_5,1596_ = 19.41, gonadal: Tukey-HSD, *P* = 0.874; larval: Tukey-HSD, *P* = 0.978). We also found dN/dS increased with greater haploid biased expression for the Warner et al. (2019) dataset (Log2 Fold β = 0.00752, *P* = 0.0058), but not the Kapheim et al. (2015) dataset (Log2 Fold β = −0.0012, *P* = 0.618).

Finally, we incorporated previous estimates of γ (γ = *2N_e_s*; where *N_e_* is the effective population size and *s* is the selection coefficient) as previously reported for honey bees (Harpur, Kent, et al. 2014). We again found that haploid-biased genes have more evidence of positive selection relative to diploid biased: 13.7% of haploid-biased genes have γ > 1 relative to 9.33% of diploid-biased genes (χ^2^= 14.53, df = 1, *P* = 0.00014). Constitutively expressed genes did not have more evidence of positive selection than haploid-biased genes (haploid 13.7% control 9.73% χ^2^ = 12.404, df = 1, *P* = 0.00042) but did not differ from diploid-biased genes (diploid 9.33% control 9.73% χ^2^ = 0.14935, df = 1, *P* = 0.6992).

### Genes Associated with Sperm Storage Are Under Strong Positive Selection

**Fig. 6.**
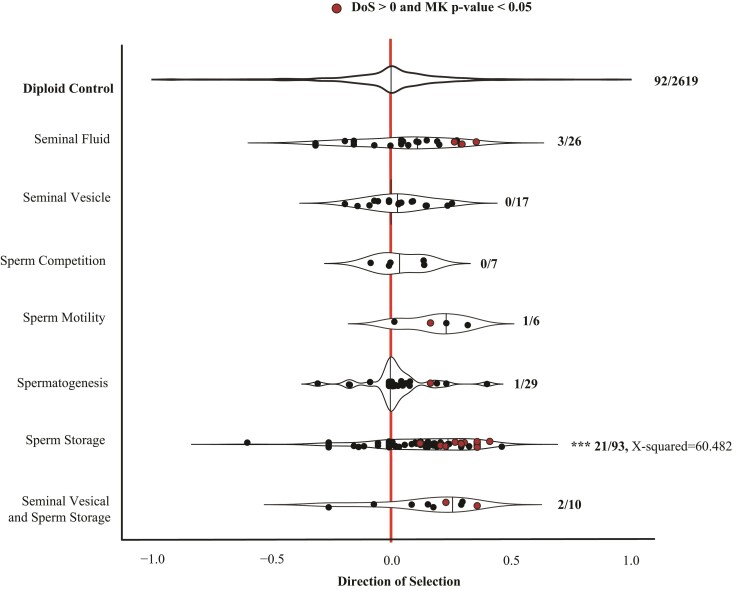
Selection on haploid-biased functional traits. Using a chi-square analysis, we found sperm storage genes had significantly more genes above a DoS of zero with a significant MK (McDonald–Kreitman test) *P*-value than the diploid-control genes. No other functional gene classes were significant. ***P < 0.0001, **P < 0.001, *P < 0.05. NS, nonsignificance.

Genes expressed in the haploid gonad had among the strongest evidence of selection across the honey bee genome. This was especially true of genes involved in sperm storage: we found that a significant proportion of genes regulating sperm storage had DoS significantly above zero compared to the diploid-biased genes (fig. 6; sperm storage 21.6%; χ^2^ = 60.482, df = 1, *P* < 0.001). Genes regulating seminal vesicles, sperm competition, sperm motility, spermatogenesis, both sperm storage and seminal vesicles, and seminal fluid did not differ from zero compared with the diploid-selected genes.

## Discussion

In modern sociogenomic studies, the male honey bee is often overlooked. Much of the attention of social insect genomic research has been aimed at understanding the role that sociality and correlates of sociality (especially those linked to female worker traits) play in driving genome-wide levels of selection and diversity (Woodard et al. 2011; Kent and Zayed 2013; Harpur, Kent, et al. 2014; Kapheim et al. 2015; Rehan and Toth 2015). Here, we explored how genes expressed by male honey bees (haploid-biased genes) are influenced by selection and how they may contribute to genome-wide patterns of diversity and divergence. In this study, we demonstrate that a significant portion of the honey bee’s protein-coding genome is expressed in the haploid stage and experiences haploid selection: at least 1,945 genes are specifically up-regulated in haploid tissue. This represents nearly 16% of the honey bee’s protein-coding genes.

Two unique features of the honey bee genome are the elevated recombination rate and strong GC-biased gene conversion (Kent et al. 2012; Liu et al. 2015; Wallberg et al. 2015). The recombination rate is high in honey bees (23cM/Mb) and varies across the genome based on predicted levels of germline methylation, GC content, genomic location, and the specific social role in which a gene is expressed (Kent et al. 2012; Liu et al. 2015; Wallberg et al. 2015). Previous work found that worker- or female-expressed genes have higher recombination rates than queen- or drone-expressed genes (Kent et al. 2012; Liu et al. 2015; Wallberg et al. 2015). The interpretation of these findings generally has been that strong selection acts on worker- or female-expressed genes and elevated recombination rates reduce the potential linkage of mutations with conflicting fitness effects among castes (Kent et al. 2012; Kent and Zayed 2013). These studies included male-biased genes from a microarray dataset derived from haploid somatic tissue (brain; Zayed et al. 2012) and found no effect of sex-expression on recombination rate. We expanded on this work by including additional male-derived tissues. We found that genes expressed in the male gonad did have elevated recombination rates and this may be associated with elevated GC content within those genes. The average GC content of genes expressed in male gonadal tissue is nearly double the average GC content of somatic tissue in males and comparable to worker-expressed genes (Kent et al. 2012; Wallberg et al. 2015). If selection across the honey bee genome favors increased recombination rates and an increase in GC-biased gene conversion (Kent et al. 2012; Wallberg et al. 2015), then haploid expressed genes in reproductive tissue are also likely targets of GC-biased gene conversion.

Evidence for positive selection fixing functional genetic variation is pervasive across the honey bee genome and previous studies highlight the importance of selection on diploid female and, specifically, worker-biased genes (Harpur, Kent, et al. 2014; Dogantzis et al. 2018). For example, studies using a Bayesian implementation of the McDonald–Kreitman test (Eilertson et al. 2012) showed worker expressed genes are more likely to experience strong selection relative those expressed in queens (Harpur, Kent, et al. 2014; Dogantzis et al. 2018). This may suggest that selection on worker-expressed genes is a major driver of adaptive change across the genome. These studies have been critically important to our understanding of how eusocial lineages evolve but they have often overlooked the haploid stage. Here, we developed (Dapper et al. 2022) and tested theory outlining how haploid- and sex-biased genes should evolve. Our model showed that asymmetries in ploidal environment and sex-biased expression offset each other, and we predicted that reproductive genes that are expressed in either a haploid- and diploid-biased fashion will have elevated levels of polymorphism and divergence when compared with constitutively expressed genes due to relaxed constraint but will not differ significantly from each other (Dapper et al. 2022). We tested these predictions across multiple tissues (reproductive, somatic, and larval) and overall found no significant support for them. Within and across tissues, haploid- and diploid-biased genes both experienced more purifying selection than expected, and there were consistent asymmetries in selection metrics between haploid- and diploid-biased genes with haploid-biased genes evolving at a significantly faster rate than diploid-biased genes. The asymmetry could result from deleterious alleles having greater fitness effects in diploid-biased genes than they do when in either haploid-biased or constitutively expressed genes. Two alternatives also exist. First, we made a monistic assumption that genes up-regulated in haploid tissues will only experience selection in that stage. Pluralism is therefore possible and a “haploid gene” in our experiment may experience selection in diploid stages as well. Second, there may be variance in the strength of selection experienced by genes expressed in haploid- or diploid-states and haploid-biased genes may experience positive selection more frequently or more strongly than diploid-biased genes.

Genes expressed in the haploid gonad generally stood out in our analyses. They have the most significant evidence of strong positive selection within out dataset based on McDonald–Kreitman tests. The McDonald–Kreitman test can provide evidence that selection (and not relaxed constraint) is the major force acting on genes with restricted expression patterns, especially those likely to experience sexual selection (Dapper and Wade 2016, 2020). Often, positive selection on sperm- or gonad-associated genes is presumed to be the result of sperm competition or PCSS (Dapper and Wade 2016, 2020). There is little evidence of either in honey bees (Baer 2005; Franck et al. 2002), and we found little evidence of positive selection specifically acting on genes associated with sperm competition based on orthologs. Instead, we find that genes associated with sperm storage have significant evidence of positive selection (fig. 6). Sperm storage is critically important to both queen and male fitness and there is likely strong selection acting on both queens and males to maintain sperm quality over the lifetime of both. Sperm maintenance is costly and trades-off with immune expression in both males and females (Sturup et al. 2013; McAfee et al. 2020). There is developing evidence of variation in honey bee sperm mortality associated with environmental features (Rangel and Fisher 2019). As well, the distribution of paternities within a colony changes significantly over time (Brodschneider et al. 2012). More research effort is certainly needed to understand and test potential mechanisms.

A fruitful continuation of this work may be to explore the role that polyandry plays in contributing to the higher rates of selection on haploid- and sperm-biased genes. Highly eusocial insects are typically polyandrous, while solitary insects within the same clade are generally monandrous (Hughes et al. 2008). The shift to polyandry by social species increases the number of mating males relative to mating females (Trivers and Hare 1976) and likely increases the efficacy of selection acting on haploid-biased genes because a higher proportion of the breeding population expresses them (Bessho and Otto 2017) (Dapper et al. 2022). We propose that honey bees provide an excellent model to explore the role of haploid selection in shaping genetic diversity and divergence, and one that should be considered in future social insect research.

## Conclusions

The often-overlooked honey bee drone provides a useful model system to understand how haploid selection and expression contribute to genome-wide levels of genetic diversity. Here, we have shown that a large fraction of the honey bee genome is expressed in the haploid state and that those genes have a unique pattern of genetic diversity and divergence relative to other genes in the genome. Ultimately, our results present an important empirical test for haploid selection hypotheses. They also provide useful insights into the evolution of social insects more broadly. There has been overwhelming attention paid to the evolution of genes expressed by diploid honey bees and their role in driving evolutionary dynamics of social insect genomes. Our results suggest that the haploid state has an important role in the evolution of arrhenotokous genomes.

## Methods

### Tissue Collection and RNA Extraction

We collected adult, flying drones (*N* = 5) and mated queens (*N* = 5) from the student apiary at Niagara College, Canada, onto dry ice. Samples were left at −80 °C until dissection. We dissected somatic (brain) and gonadal (testes and associated glands; ovaries) tissues from each sample following previously-established protocols (Niu et al. 2014; Vleurinck et al. 2016). In brief, samples were dissecting in chilled RNA*later*^™^ ICE (ThermoFischer Scientific, USA) and dissected tissue was immediately placed into Trizol. Several drone tissues undergo limited endoreplication and are effectively diploid (Aron et al. 2005) because of this, we chose brain tissue as a representative somatic tissue. RNA was extracted from the tissues using the Invitrogen Trizol Protocol and purified using a Qiagen RNeasy mini kit (Qiagen, USA).

### cDNA Library Generation and Illumina Sequencing

The RNAseq library was prepared using the Illumina TruSeq stranded mRNA sample preparation kit according to the manufacturer’s guidelines. Poly-A containing RNA was purified from total RNA using poly-T oligo attached magnetic beads after which mRNA will be fragmented and reverse transcribed to first strand cDNA using random primers. The cDNA fragments were ligated to adapters and purified cDNA libraries enriched with PCR. All sequencing was performed using Illumina HiSeq 2500 sequencing technology producing 150-bp length paired-end reads.

### Gene Expression Analysis

In addition to the gonadal and somatic tissue above, we also included data from a recently published study on haploid- and diploid- fifth instar larval gene expression (NCBI; BioProject PRJNA260604). After trimming of Illumina adaptors using Trimmomatic (Bolger et al. 2014), we pseudo-aligned reads to the most recent version of the honey bee transcriptome (NCBI; Amel_HAv3.1) using Kallisto (Bray et al. 2016). Following pseudo-alignment, we removed from each sample any remaining counts associated with rRNA genes and analyzed the resulting count data with DeSeq2 (Anders 2010). Within DESeq2, we removed any gene with fewer than ten read counts across all samples and then ran pairwise models to identify up-regulated genes in each sex and tissue. This analysis allows us to identify both tissue and sex specific genes. Constitutively expressed genes were genes up-regulated in both sexes. We did this separately for the larval and adult data sets. The larval data used a single pairwise comparison between female larvae, male larvae, and worker larvae, which found genes uniquely expressed in each larval caste. We then proceeded with gene lists for both queens and workers. The overlap between various gene sets was visualized using *UpsetR* (Conway et al. 2017). The pairwise comparison was done for each sample type and genes were included if: 1) the gene was up-regulated in each comparison and 2) if they were significantly differentially expressed following a False Discovery Rate (FDR) correction of *P* < 0.01.

### Calculating Population Genetic Statistics

To calculate the population genetic parameters used in this study, we used a recent population genomic data set for an African honey bee subspecies and for *A. cerana,* a sister species to *A. mellifera* (Harpur, Kent, et al. 2014). We used the African subspecies because they are less likely to have experienced selection due to management practice. We re-aligned all 11 *A. melifera scutellata* samples and the single *A. cerana* sample from this study to the latest honey bee genome (NCBI; Amel_HAv3.1) following the protocol outlined in that study (Harpur, Kent, et al. 2014). In brief, we aligned the single-ended reads using BWA (Li and Durbin 2010) and re-aligned Stampy (Lunter and Goodson 2011). We then removed duplicate reads with Picard tools. We used GATK (Depristo et al. 2011) for Base Recalibration using a set of previously identified haploid-called SNPs (Harpur et al. 2019). Once recalibrated, we used GATK Haplotypecaller and CombineGVCFs for joint genotyping across all samples.

We calculated both πNS and πS for the 11 African honey bee samples, using SNPGenie (Nelson et al. 2015) for all sites with a minimum allele frequency greater than 0.01. We also followed a previously established protocol (Harpur, Kent, et al. 2014) to count the number of nonsynonymous and synonymous polymorphic and fixed differences within *Apis*. We identified mutations as synonymous or nonsynonymous with SnpEff (Cingolani et al. 2012). We calculated the DoS (Stoletzki and Eyre-Walker 2011) between *A. cerana* and *A. mellifera* as DoS = Dn/(Dn + Ds) − Pn/(Pn + Ps) where Dn and Ds represent the number of fixed nonsynonymous (Dn) and synonymous (Ds) mutations between the two species and Pn and Ps represent the number of polymorphic nonsynonymous (Pn) and synonymous (Ps) mutations within species. For each gene, we also estimate the significance of a standard McDonald–Kreitman test (McDonald and Kreitman 1991) using a χ^2^ test. DoS measures the direction and extent of selection on a given gene and is positive when there is evidence of adaptive evolution, zero when neutral, and negative when slightly deleterious mutations are segregating within a population (as would occur with demographic events).

We estimated the recombination rate of each gene using a linkage map developed for the honey bee genome (Solignac et al. 2007). Because this map was designed for a previous version of the honey bee genome, we first used BLAST to identify the locations of each of the ∼2,000 markers included in this study on the updated honey bee genome, as has been previously described (Wallberg et al. 2015). We used this updated linkage map to identify the recombination rate experienced by all genes in the genome using the MareyMap package in R (Rezvoy et al. 2007) and extracting the estimated recombination rate for a given gene region.

### Model

To evaluate whether there is evidence that selection acts differently on these classes of genes, it is necessary to first establish the null expectation for the relative amount of polymorphism and divergence we expect to observe if there are no differences in strength or DoS (selection coefficient). Ploidy, sex-specific expression, and putative involvement in PCSS are all confounding factors that determine how effectively selection removes deleterious mutations and fixes beneficial ones. Here we apply the theoretical expectations derived in Dapper et al. (2022) for arrhenotokous species with diploid females and haploid males. The null expectation for male- or female-biased genes is expected to be two-fold that observed in constitutively expressed genes, due to relaxed selection. We found the average Pn/Ps of control, constitutively expressed genes to be approximately 0.10 for control genes, which gives an expected Pn/Ps of 0.20 for sex biased loci.

The null expectation for divergence (dN/dS) depends upon the product of the effective population size and the average selective effect (*N_e_s*). We observed an average dN/dS of 0.11 among control, constitutively expressed loci, allowing us to estimate an average *N_e_s* = −1.396 for the honey bee genome. Using this estimated value of *N_e_s,* we found the null expectation of the average dN/dS of sex biased genes to be 0.37 (Dapper et al. 2022).

The potential for these sex biased loci to predominantly function in sperm competition or cryptic female choice raises the prospect they may be subject to PCSS. The efficacy of selection on genes that function in PCSS is positively correlated with the harmonic mean number of mates per female (*H*). We estimate H is quite high among honey bees (*H* = 14) and as a result, the null expectation is for such genes is not expected to be much higher than that expected for sex-specific loci that are not involved in PCSS.

### Function of Haploid-Selected Genes

To functionally characterize genes that are most likely to be expressed in haploid males we identified genes specific to male phenotypes. We extracted gene identifiers from studies on honey bee seminal fluid (Baer et al. 2009), seminal vesicles (Collins et al. 2006) and stored sperm (Collins et al. 2006; Poland et al. 2011). We further identified genes associated with or involved in spermatogenesis, sperm competition, and sperm motility by extracting *Drosophila* genes associated with each of these Gene Ontology categories from FlyBase and identifying their BLAST Best Matches in honey bee genes. We cross-referenced these two lists with expression data above to create a set of genes that are 1) expressed in haploid tissue and 2) previously associated with male traits.

### Data Analysis

All analyses were performed in R v 3.1.3 (Team 2013) and were parametric unless otherwise stated. We performed all analyses with and without GC content as a covariate.

## Data Availability

All data generated for this study have been deposited with NCBI SRA (PRJNA689223).
